# NMR-based metabolic profiling and comparison of Japanese persimmon cultivars

**DOI:** 10.1038/s41598-019-51489-y

**Published:** 2019-10-18

**Authors:** Shoraku Ryu, Tomonari Muramatsu, Kazuo Furihata, Feifei Wei, Masanori Koda, Takuya Miyakawa, Masaru Tanokura

**Affiliations:** Department of Applied Biological Chemistry, Graduate School of Agricultural and Life Sciences, The University of Tokyo, 1-1-1 Yayoi, Bunkyo-ku, Tokyo 113-8657 Japan

**Keywords:** Metabolomics, Analytical biochemistry

## Abstract

Persimmons are a traditional, autumnal, and healthy fruit commonly consumed in Japan and East Asia based on the saying, “a persimmon a day keeps the doctor away.” The differences in metabolites among five major Japanese persimmon cultivars were investigated using a nuclear magnetic resonance (NMR)-based metabolomics approach. By using a broadband water suppression enhanced through *T*_1_ effects (WET) method for the sensitive detection of minor metabolites, better discrimination among cultivars and more informative details regarding their metabolic differences have been achieved compared to those achieved in conventional ^1^H NMR sequences. Among the nonastringent cultivars analyzed, the Taishu cultivar has the highest abundance of amino acids. The Matsumotowase-Fuyu cultivar contains ethyl-β-glycosides as characteristic components, which may relate to fruit softening. Citric acid concentration is higher in Maekawa Jiro than in other nonastringent cultivars. Among the two astringent cultivars analyzed, ethanol was significantly higher in Hiratanenashi than in Yotsumizo, which indicates different reactivity during deastringency treatments. The present study proposes an efficient and relatively quantitative metabolomics approach based on broadband WET NMR spectra.

## Introduction

In recent years, food science has increasingly developed, transitioning from focusing on specific components to exploring the entire composition of foods and connecting differences in composition to varietal characteristics, the nutritional value of cultivars, the physical benefits of foods, *etc*. This improvement has been achieved by applying advanced analytical methodologies and omics tools to food science. There is a new discipline termed “Foodomics”, which is a new approach to studying food through the integration of omics technology^1,2^. Among omics technologies, nuclear magnetic resonance (NMR) spectroscopy is a rapid and unbiased technology that can be used for the simultaneous analysis of components in foods as an entire mixture with minimum destruction of samples. In addition, the capacity for highly reproducible results and the quantitative properties of NMR spectroscopy are important advantages^3,4^. NMR has increasingly become an attractive tool in metabolomics analysis and has been combined with multivariate data analysis such as principal component analysis (PCA) and partial least-squares discriminate analysis (PLS-DA)^5–8^. This approach has been used to identify differences among varieties of foods and provides robust support for determining the origin of a food, the quality of cultivar selection, taste evaluation, *etc*^9–18^.

*Diospyros kaki*, better known as the Japanese persimmon, is the most widely cultivated species of the *Diospyros* genus. Persimmons are recognized as an outstanding source of biologically active components that exhibit many health benefits, such as antioxidant behavior, radical scavenging activity, antihypertensive activity, and antiatherosclerosis activity^19–23^. Persimmon cultivars are classified broadly into two categories: astringent cultivars and nonastringent cultivars. An astringent cultivar is not suitable for human consumption at the time of harvest due to the high amount of soluble tannins in the fruit. It is suitable for human consumption after artificial removal of its astringency or once it is overripe or dried^24–26^, while a nonastringent cultivar is edible at the time of harvest. It is thought that the seeds in persimmons have the ability to remove astringency during the development of the fruit by turning soluble tannins into insoluble tannins^27–29^. Approximately 1,000 varieties are estimated to be grown in Japan. The composition of biological components varies among different cultivars^30^. However, despite the variety of Japanese persimmon cultivars, there are limited reports on the compositional differences among persimmon cultivars^23,31^. Furthermore, no report has produced a metabolic profiling characterization of different cultivars of Japanese persimmons using NMR.

In the present study, we provide detailed and comprehensive information regarding the compositional differences among five leading commercial Japanese persimmon cultivars using NMR spectroscopy combined with multivariate data analysis. The aim of this study is to provide an efficient and relatively quantitative approach to characterize the compositional differences in different persimmon cultivars. The five cultivars include three nonastringent persimmons, ‘Taishu (TS)’, ‘Matsumotowase-Fuyu (MF)’ and ‘Maekawa Jiro (MJ)’, as well as two astringent cultivars, ‘Hiratanenashi (HN)’ and ‘Yotsumizo (YM)’. Since detection of the minor components of persimmons are hindered by the main components (sugars) and are often undetectable or detected with very poor signal-to-noise (S/N) ratios in NMR analysis, the broadband water suppression enhanced through *T*_1_ effects (WET) method, which was proposed in our previous study^32^, was utilized to unambiguously identify minor components.

## Results and Discussion

### ^1^H NMR spectra and broadband WET spectra of five japanese persimmon cultivars

**Figure 1:**
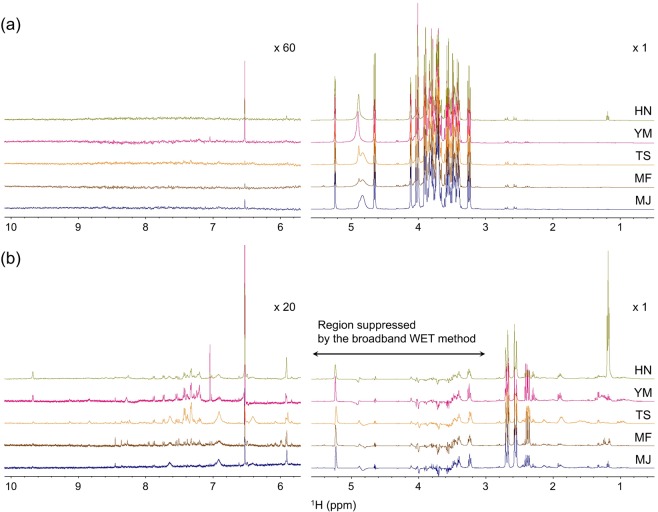
Conventional ^1^H NMR spectra **(a)** and broadband WET NMR spectra **(b)** of five persimmon cultivars. HN, YM, TS, MF and MJ represent Hiratanenashi, Yotsumizo, Taishu, Matsumotowase-Fuyu and Maekawa Jiro, respectively.

Figure 1 shows the ^1^H NMR spectra of juice from five different persimmon cultivars. The signal patterns of ^1^H NMR spectra for all five cultivars were similar. The spectral region between 3.10 and 5.40 ppm contained signals mainly from sugars (glucose, fructose and sucrose), which are the dominant components in all cultivars (Fig. 1a). As shown in the expanded spectra of the low-field region (6.0–10.0 ppm), few peaks were observed in the low-field region for all cultivars. The broadband WET method^32^ was successfully used as a new method for qualitatively and quantitatively observing relatively minor components in fruit juice via the simultaneous suppression of strong signals derived from major components (water and sugars) and provision of high-throughput information at a high S/N ratio for minor components in a complex mixture. As expected, new signals were detected in the low-field region with the same number of scans (128 scans) in the broadband WET spectra (Fig. 1b).

Components were assigned according to a previous report^33^. Signals in the region from 0.5 ppm to 3.0 ppm were derived from protons of free amino acids including alanine (δ 1.47), 4-aminobutanoic acid (GABA) (δ 1.89, δ 2.29), aspartic acid (δ 2.66, δ 2.81), citrulline (δ 1.51, δ 1.57, δ 3.75), glutamic acid (δ 2.05, δ 2.11, δ 2.35), glutamine (δ 2.12, δ 2.43), isoleucine (δ 0.93, δ 1.24, δ 1.47, δ 1.98), leucine (δ 0.95, δ 1.71, δ 1.73), threonine (δ 1.31) and valine (δ 0.97, δ 1.02, δ 2.27) as well as organic acids including citric acid (δ 2.61, δ 2.72) and malic acid (δ 2.39, δ 2.69). The signals in the region from 6.0 ppm to 10.0 ppm were considered to be derived from acetaldehyde (δ 9.67), adenosine (δ 6.06, δ 8.31), fumaric acid (δ 6.52), trigonelline (δ 8.09, δ 8.84, δ 9.12), and uridine (δ 7.87). In this region, the broad signals were assigned to the amino protons from glutamine (δ 6.92, δ 7.62) and citrulline (δ 6.38), which were confirmed by analysis of a sample spiked with standard compounds. A portion of these assignments was also confirmed by the statistical method STOCSY^34^ (Supplementary Fig. S1).

### Cultivar discrimination using broadband WET spectra

**Figure 2:**
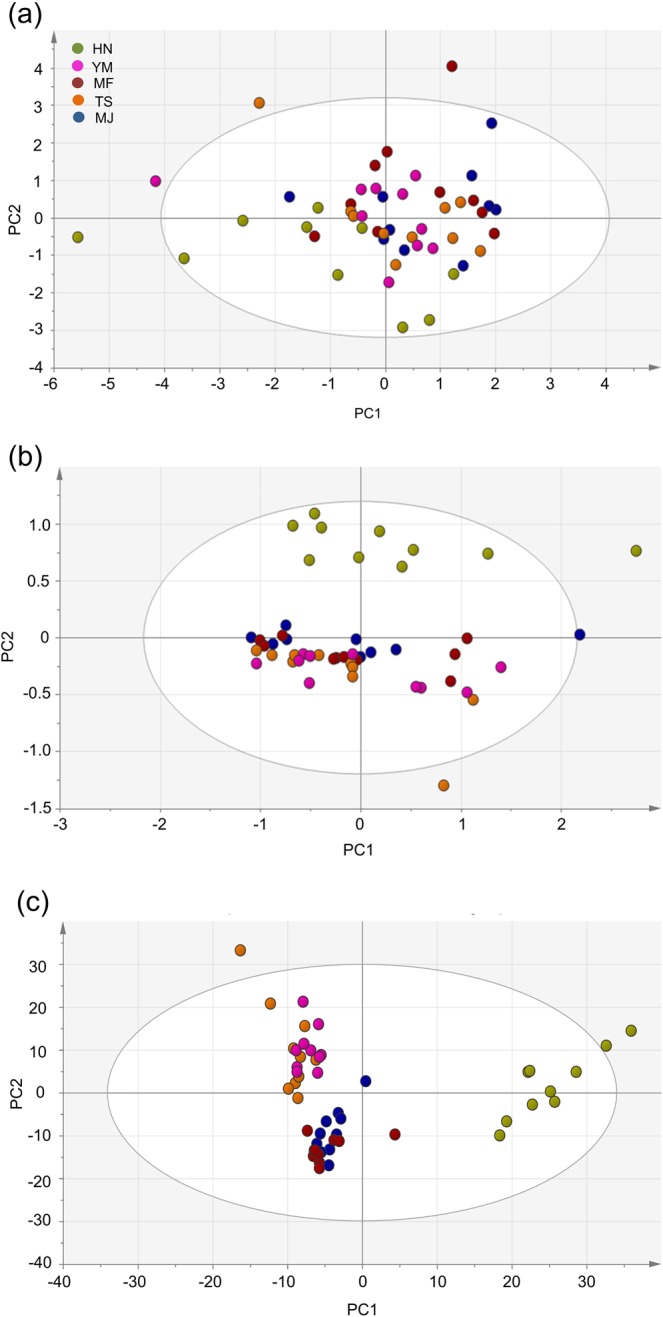
PCA score plots of five cultivars derived from **(a)** conventional ^1^H NMR spectra with the exclusion of the water suppression region between 4.5 ppm and 5.2 ppm. **(b)** Conventional ^1^H NMR spectra with the regions of water and sugars (between 3.0 ppm and 5.6 ppm) excluded. **(c)** Broadband WET spectra with the water and sugar saturation regions within 3.0 to 5.6 ppm excluded. HN, YM, TS, MF and MJ represent Hiratanenashi, Yotsumizo, Taishu, Matsumotowase-Fuyu and Maekawa Jiro, respectively.

To investigate the variation in overall composition, PCA was performed on the dataset of five cultivars (10 samples of each), and the results of the multivariate data analysis for the ^1^H NMR and broadband WET spectra were compared. The score plots are shown in Fig. 2.

Initially, the conventional ^1^H NMR spectra, excluding only the water suppression region (4.5 ppm to 5.2 ppm), showed no statistically significant differences among cultivars in the score plot (Fig. 2a). Then, PCA was applied to the conventional ^1^H NMR spectra, except for the regions of water and sugar suppression (3.0 ppm to 5.6 ppm). The score plot showed that HN clustered far from the other four cultivars according to PC2 (Fig. 2b), indicating that this cultivar was significantly different from other cultivars and that the other four cultivars were similar in metabolite composition. This result revealed that an improvement in discrimination among cultivars was achieved by excluding the sugar region in addition to the water suppression region of ^1^H NMR spectra before applying PCA. Such differences among cultivars might be dictated by metabolites other than sugars because concentrations of sugars are more affected by ripeness and deastringency treatments than by cultivars. However, PC2 explained only a low percentage (17.2%) of the total variance for all five cultivars, which indicates that the discrimination result is not sufficiently informative.

PCA was then applied to the broadband WET spectra, excluding both the water and sugar saturation regions within 3.0 ppm to 5.6 ppm. The score plot (Fig. 2c) showed that the first two PCs (PC1 and PC2) explain 33.9% and 26.2% of the total variance obtained for fruit cultivars, respectively. HN was clearly separately clustered from the other 4 cultivars according to PC1, and cultivars YM and TS were separately clustered from cultivars MF and MJ according to PC2. The results showed that discrimination among cultivars was improved by using broadband WET spectra combined with the exclusion of the sugar and water regions. This improved discrimination result suggested that minor components might be responsible for cultivar discrimination because more minor components were detected in the broadband WET spectra than in the conventional ^1^H NMR spectra.

**Figure 3:**
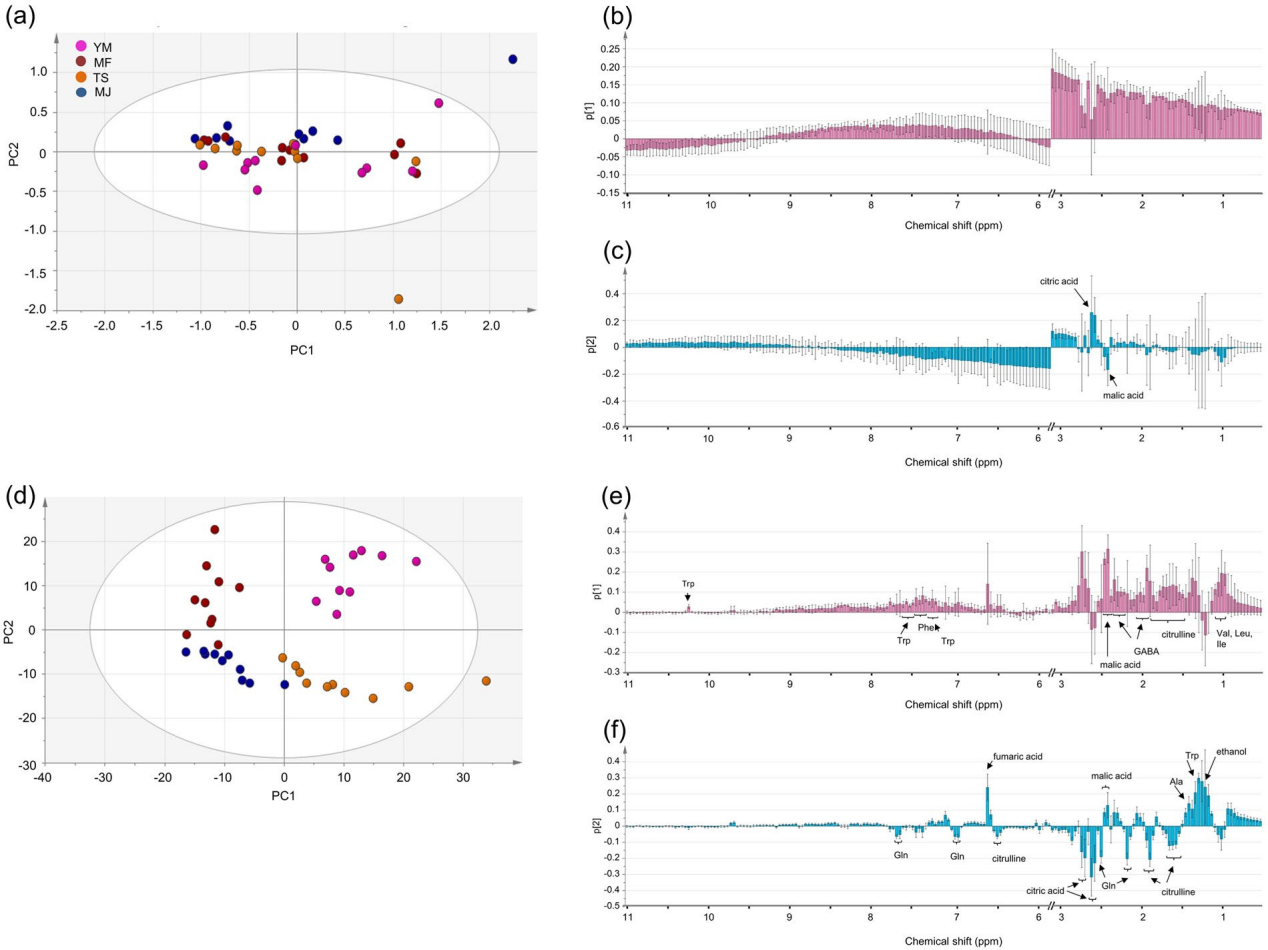
**(a)** PCA score plot of four cultivar samples derived from conventional ^1^H NMR spectra with the regions for water and sugars (between 3.0 ppm and 5.6 ppm) excluded. Loading plots for **(b)** PC1 and **(c)** PC2 of PCA analysis of conventional ^1^H NMR spectra **(a)**. **(d)** PCA score plot of four cultivar samples derived from broadband WET spectra with the water and sugar saturation regions within 3.0 to 5.6 ppm excluded. Loading plots for **(e)** PC1 and **(f)** PC2 of PCA analysis of broadband WET spectra **(d)**. YM, TS, MF and MJ represent Yotsumizo, Matsumotowase-Fuyu, Taishu and Maekawa Jiro, respectively.

To further explore whether multivariate data analysis combined with broadband WET spectra can extract the features of similar cultivars more effectively than conventional ^1^H NMR spectra, another PCA analysis was performed using only four cultivars (YM, TS, MF and MJ) and excluding HN. The PCA score plots for conventional ^1^H NMR and broadband WET spectra are shown in Fig. 3, both of which exclude the regions of water and sugars (3.0 ppm to 5.6 ppm). The PCA results for the conventional ^1^H NMR spectra showed almost no separation among cultivars, although PC1 and PC2 explained 59.6% and 14.5% of the variance, respectively. In contrast, all four cultivars with similar metabolite compositions separately clustered on the score plot of the first two principle components derived from the broadband WET spectra, where over half of the total variance was explained by PC1 (37.2%) and PC2 (29.6%). The results showed that broadband WET spectra combined with the exclusion of the water and sugar regions exhibited good differentiation among cultivars and were very useful for investigating the differences in metabolites that are intrinsic to persimmon cultivars. The discrimination between cultivars, in PCA analysis, obtained by the use of the broadband WET NMR data could result from the minor compounds detected by the method, which contribute to the loadings for PC1 and PC2 (Fig. 3e,f). On the other hand, only citric acid and malic acid appeared in the loading plot for the analysis using the conventional NMR data (PC2, Fig. 3c).

### Comparison of metabolite differences between astringent and nonastringent cultivars

OPLS-DA was used to isolate the metabolites responsible for the differences between astringent and nonastringent cultivars. The goodness-of-fit parameter *R*^2^*x* and the predictive ability parameter *Q*^2^ were also compared. *R*^2^*x* varies between 0 and 1, where 1 indicates a perfect fit between the model and the data. When *Q*^2^ is greater than 0.5, the model is considered to have good predictability, and if *Q*^2^ is greater than 0.9, then the model is considered to have excellent predictability.

**Figure 4:**
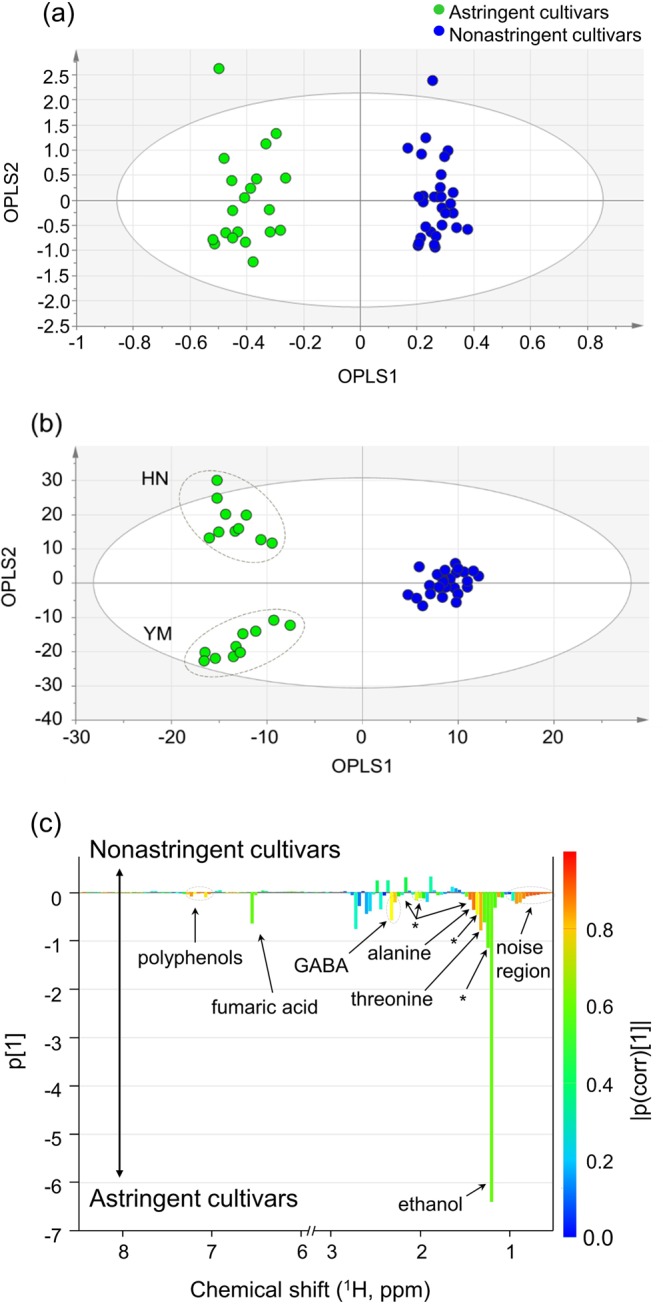
**(a)** OPLS-DA score plot for five cultivars derived from ^1^H NMR spectra with the regions for water and sugars (between 3.0 ppm and 5.6 ppm) excluded. The variances were 8.8% and 54.4% for OPLS1 and OPLS2, respectively. The goodness-of-fit parameters *R*^2^*x* and *R*^2^*y* were 0.940 and 0.966, respectively, and the *Q*^2^ value was 0.761. **(b)** OPLS-DA score plot and **(c)** OPLS-DA loading plot of five cultivars derived from broadband WET spectra with the water and sugar saturation regions within 3.0 to 5.6 ppm excluded. HN and YM represent the Hiratanenashi and Yotsumizo cultivars, respectively. Asterisks denote unknown components. The variances were 23.1% and 27.5% for OPLS1 and OPLS2, respectively. The goodness-of-fit parameters *R*^2^*x* and *R*^2^*y* were 0.870 and 0.965, respectively, and the *Q*^2^ value was 0.936.

Based on the results of PCA presented above, OPLS-DA was applied to the broadband WET spectra without the water and sugar saturation regions from 3.0 ppm to 5.6 ppm. For comparison, OPLS-DA was also applied to conventional ^1^H NMR spectra without the regions attributed to water and sugars (between 3.0 ppm and 5.6 ppm). The OPLS-DA score plots are shown in Fig. 4. In the score plot generated from the ^1^H NMR spectra (Fig. 4a), astringent and nonastringent cultivars were discriminated with a total variance of 63.2% (8.8% for OPLS1 and 54.4% for OPLS2). The goodness-of-fit parameters *R*^2^*x* and *R*^2^*y* were 0.940 and 0.966, respectively, and the predictive ability parameter *Q*^2^ value was 0.761.

The OPLS-DA score plot derived from broadband WET spectra (Fig. 4b) showed a total variance of 50.6% (23.1% for OPLS1 and 27.5% for OPLS2). The OPLS-DA model revealed *R*^2^*x*, *R*^2^*y*, and *Q*^2^ values of 0.870, 0.965, and 0.936, respectively, all of which indicate excellent fit and prediction abilities for this OPLS regression model. Moreover, HN and YM were separately grouped on the OPLS-DA score plot in addition to the discrimination between astringent and nonastringent cultivars.

The corresponding OPLS-DA loading plot illustrates metabolite differences among cultivars. The loading plot for OPLS1 of OPLS-DA modeled using broadband WET spectra (Fig. 4c) showed that the metabolites responsible for the differentiation between astringent and nonastringent cultivars were ethanol, fumaric acid, threonine, GABA, alanine and some unknown metabolites corresponding to several characteristic signals in astringent cultivars, such as the broad signals at 1.10–1.14 ppm, 1.26–1.30 ppm, and 2.02–2.30 ppm and the singlet signals at 7.05–7.09 ppm and 7.21–7.29 ppm that are presumed to be signals from polyphenols. A more informative characterization of metabolic differences among cultivars was achieved by comparing broadband WET spectra with the ^1^H NMR spectra. Threonine, GABA, and alanine can be more clearly detected (with a good S/N ratio) in the broadband WET spectra than in the conventional ^1^H NMR spectra, and the singlet signals at 7.05–7.09 ppm and 7.21–7.29 ppm can only be observed in broadband WET spectra.

### Comparison of metabolite differences between pairs of cultivars

Since broadband WET spectra represented a suitable methodology for studying cultivar differentiation, OPLS-DA was applied to spectra of the five cultivars, and the metabolites that are mainly responsible for the separation were determined.

**Figure 5:**
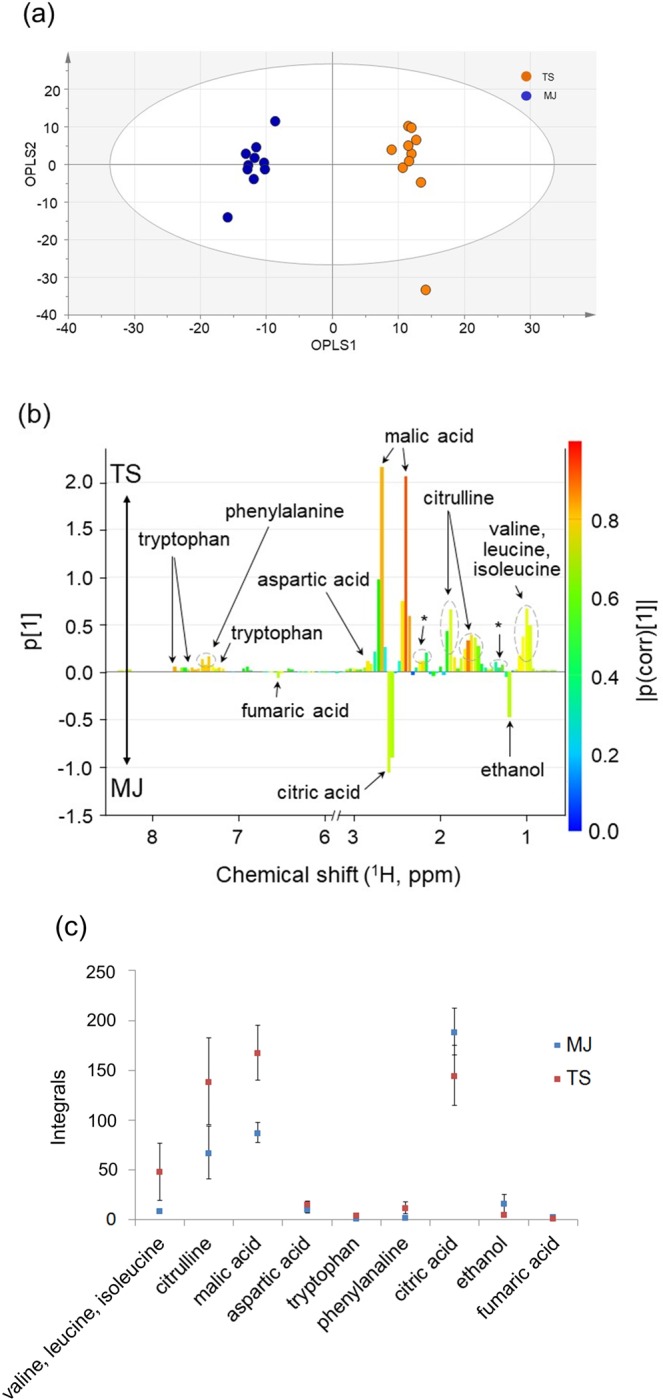
**(a)** OPLS-DA score plot and **(b)** OPLS-DA loading plot of two nonastringent cultivars (TS and MJ) derived from broadband WET spectra with the water and sugar saturation regions from 3.0 to 5.6 ppm excluded. The variances were 45.6% and 28.7% for OPLS1 and OPLS2, respectively. The goodness-of-fit parameters *R*^2^*x* and *R*^2^*y* were 0.804 and 0.982, respectively, and the *Q*^2^ value was 0.945. **(c)** The integral intensities of biomarkers in TS and MJ cultivars. TS and MJ represent Taishu and Maekawa Jiro, respectively. Asterisks denote unknown components. The NMR spectrum of ten persimmon fruits for each of the cultivars was measured individually (*n* = 10, Error bar: ±standard deviation).

For the TS and MJ cultivars, the loading plot for OPLS1 (Fig. 5) showed relevant metabolites for the differentiation of the two groups. Some amino acids and organic acids were analyzed as variables of the loading plot and were considered to contribute to the differences between the TS and MJ cultivars in OPLS1. As shown in Fig. 5b, valine, leucine, isoleucine, citrulline, malic acid, aspartic acid, tryptophan, and phenylalanine were observed as typical components of TS. In contrast, ethanol, citric acid and fumaric acid were detected as biomarkers in MJ. The integral values are shown in Fig. 5c.

Additionally, comparisons were performed between TS and MF. The total variance of the separation between the two cultivars is 0.763 (0.564 for OPLS1 and 0.199 for OPLS2). In TS, amino acids (valine, leucine, isoleucine, threonine, citrulline, GABA, glutamine, phenylalanine, and tryptophan) and malic acid were obviously higher than in MF. In addition, signals at 1.148–1.22 ppm were found to be biomarkers of the MF cultivar (Supplementary Fig. S2). These peaks were partially assigned to a series of ethyl-β-glycosides with the aid of 2D NMR spectra (^1^H-^1^H DQF-COSY, ^1^H-^1^H TOCSY, ^1^H-^13^C CT-HMBC, and ^1^H-^1^C HSQC), which are shown in Supplementary Fig. S3. Ethyl moieties were confirmed by cross-peaks in the ^1^H-^1^H DQF-COSY spectrum, which were 1.22 (t)/3.71 ppm, 1.22 (t)/3.95 ppm, 1.148 (t)/3.75 ppm, 1.154 (t)/3.75 ppm, and 1.17 (t)/3.65 ppm. The chemical shifts of –CH_3_ were obtained as 1.22/17.05 ppm and 1.154/17.71 ppm by the ^1^H-^13^C HSQC spectrum and overlapped with the cross-peaks at 1.148/20.36 ppm and 1.17/19.70 ppm. From the ^1^H-^13^C CT-HMBC spectrum, the cross peaks at 1.22/69.16 ppm, 1.148/82.63 ppm, 1.154/73.65 ppm, and 1.17/60.18 ppm were identified and interpreted as representing the coupling between the protons of –CH_3_ and the carbons of –CH_2_ in the ethyl moieties. Furthermore, –CH_2_ in the ethyl moieties was shown to be linked to a glycoside via a glycosidic bond by the cross-peaks at 4.46/69.16 ppm, 4.52/82.63 ppm, and 4.49/73.65 ppm in the ^1^H-^13^C CT-HMBC spectrum, which were considered to be derived from the anomeric protons. However, a correlation between 60.18 ppm and any saccharides was not observed in the ^1^H-^13^C HMBC spectrum. Moreover, in the ^1^H-^1^H TOCSY spectrum, the *J*-coupled spin systems were identified as 4.46/3.25/3.46/3.38 ppm, 4.52/3.26/3.48/3.39 ppm, and 4.49/3.31/3.48/3.38 ppm. The cross-peak at 4.46/3.25 ppm was also detected in the ^1^H-^1^H DQF-COSY spectrum, indicating that the two protons coupled with each other. The spin systems were very similar to glucose and were considered to be glycosides derived from saccharides. It was reported that the MF cultivar loses its firmness quickly after harvest, which relates to the degradation of cell wall polysaccharides by polygalacturonase (PG)^35^. The glycoside may originate from the degradation of cell wall polysaccharides such as polyuronide and xyloglucans during softening^36^. Yoshioka *et al*. suggested that de-esterification of polyuronides occurs with a high degree of methoxylation during the softening of apples and pears^37^. In addition, the sugar component ethyl-β-glucopyranoside was found in sea buckthorn fruit^38^ and has a very similar chemical shift to that observed in the results of this study. Therefore, we tentatively assigned those signals to ethyl-β-glycosides, possibly in the form of monosaccharides or polysaccharides.

A comparison between MF and MJ cultivars was also performed. The loading plot (Supplementary Fig. S4) for OPLS1 showed that the variables contributing to the separation were amino acids, organic acids (malic acid and citric acid), and ethyl-β-glycosides. The comparison of the integral values showed that the largest differences in integral values were those of ethyl-β-glycosides and malic acid, which were obviously abundant in the MF cultivar. In contrast, citric acid was higher in the MJ cultivar.

From the results presented above, amino acids are higher in TS than in the other two cultivars (MF and MJ), and ethyl-β-glycosides are the most abundant characteristic component in the MF cultivar. In addition, citric acid is higher in the MJ cultivar than in the other two cultivars. Variations in these metabolites may be responsible for variations in flavor, texture, ripening, and softening among cultivars. The results of the metabolite-based analyses provide meaningful data for Japanese persimmon studies and the application of this method combined with other assessments such as a quality assessment can help with the selection or modification of better quality or nutritionally important cultivars.

OPLS-DA was also performed on the two astringent cultivars for comparison (Supplementary Fig. S5). Citric acid in the HN cultivar was higher than in the YM cultivar. However, the YM cultivar included more malic acid than the HN cultivar. In the HN cultivar, ethanol was significantly higher than in the YM cultivar, and the signals at 0.86–0.98 ppm and 1.38–1.42 ppm that were unassigned (indicated with asterisks in the figure) were the marker components in the YM cultivar. Yamada *et al*. reported the metabolic pathway for acetaldehyde and ethanol production following CO_2_ and ethanol deastringency treatment, and a significant variety of treatment reactions were observed among the cultivars^39^. The results suggested that the two cultivars might have different enzyme activities or metabolic pathways during CTSD treatment.

## Conclusions

Differences in metabolites among five major Japanese persimmon cultivars were investigated using broadband WET spectra combined with multivariate data analysis. The PCA models clearly demonstrated that cultivar discrimination of Japanese persimmons by ^1^H NMR spectra can be improved by removing major bulk signals (water and sugars). Improved discrimination and more informative details regarding the metabolic differences among cultivars were achieved using broadband WET spectra compared to those achieved in conventional ^1^H NMR sequences. The same strategy can also be applied to other fresh foods, especially those that are predominated by some major components. Some amino acids (citrulline, GABA, tryptophan, and phenylalanine), organic acids (malic acid and citric acid), and ethanol were determined to be biomarkers responsible for metabolite differences among the cultivars in this study. The results provide meaningful data for Japanese persimmon studies, and in the future, this approach combined with quality or other assessments will help in the selection or modification of better or nutritionally important cultivars.

## Materials and Methods

### Sample preparation

Three nonastringent (TS, MF, and MJ) and two astringent (HN and YM) persimmon cultivars were supplied by the National Agriculture and Food Research Organization (Hiroshima, Japan). TS is a new cultivar that was released in 1995, which is a mid-ripening type with very large fruits (approximately 400 g) of excellent quality (very soft with juicy flesh). Fruit can develop shallow and concentric cracks in the skin, leading to a high sugar content in the flesh beneath the cracks^40,41^. MF is an early-ripening cultivar selected from the ‘Fuyu’ persimmon and has a firm and crunchy flesh^35^. MJ is also an early-ripening cultivar and has sweet, mild, firm and flavorful flesh. HN and YM are two well-known astringent persimmon cultivars. HN is a high-quality cultivar after it is treated to remove astringency, and YM is often used to prepare dried persimmons^42^. Ten fruits from each of the five cultivars were collected from the same field in mid-November of same year. The two astringent persimmons were subjected to the experiments after constant temperature short duration (CTSD) treatment to remove astringency.

The fruits were ground into pulp and then centrifuged at 13,000 × g for 10 min at 4 °C. The supernatants were used as persimmon juice samples. The juices were stored at −30 °C until NMR analysis. Persimmon juice (630 μL) was mixed with 70 μL 0.1 M phosphate buffer (pH 6.5) prepared with D_2_O (99.9%, Shoko Co., Ltd., Tokyo, Japan). TSP-*d*_4_ solution (35 mM, Sigma-Aldrich Co. LLC., St. Louis, MO) was added to the sample as both a chemical shift and quantitative reference to a final concentration of 0.1 mM. EDTA (0.5 M, Dojindo, Kumamoto, Japan) was added to the sample to a final concentration of 1.5 mM to capture the paramagnetic ions that cause broadening of NMR signals. The mixture was then transferred into a 5-mm NMR tube.

### NMR spectroscopy

NMR spectra were obtained at 20 °C on a Varian Unity INOVA-500 spectrometer (Agilent Technologies, Ltd., Santa Clara, CA) for the ^1^H, ^1^H broadband WET, ^13^C, ^1^H-^13^C HSQC, ^1^H-^1^H TOCSY, and ^1^H-^1^H DQF-COSY spectra, and a Varian Unity INOVA-600 spectrometer equipped with a cryogenic probe (Agilent Technologies, Ltd., Santa Clara, CA) was used for the ^1^H-^13^C CT-HMBC spectrum.

The ^1^H NMR spectra were recorded with a spectral width of 8,000 Hz, the number of data points was 64 k, the number of scans was 128, the acquisition time was 2.048 s, and the delay time was 5.0 s. The water signal was suppressed by a presaturation method.

The ^13^C NMR spectra were recorded with a spectral width of 31,422 Hz, the number of data points was 16 k, the number of scans was 52,896, the acquisition time was 1.043 s, and the delay time was 2.0 s.

The acquisition parameters of the broadband WET method^27^ were as follows: spectral width, 8,000 Hz; number of data points, 64 k; number of scans, 128; acquisition time, 2.048 s; and delay time, 5.0 s. The pulse length of the e-Burp pulse in the broadband WET method was 3.88 ms.

The acquisition parameters of total correlation spectroscopy (TOCSY) were as follows: spectral width, 6,700 Hz (F1) and 6,700 Hz (F2); number of data points, 512 (F1) and 2,048 (F2); number of scans, 32; acquisition time, 0.341 s; delay time, 2.0 s; field strength of MLEV-17 spin-lock pulse, 7.1 kHz; length of trim pulse, 2 ms; and mixing time, 80 ms.

For double-quantum filtered correlation spectroscopy (DQF-COSY), the spectral widths were 6,000 Hz (F1) and 6,000 Hz (F2), the number of data points were 512 (F1) and 2,048 (F2), the number of scans was 16, the acquisition time was 0.341 s, and the delay time was 2.0 s.

The acquisition parameters of the ^1^H–^13^C heteronuclear single-quantum correlation spectroscopy (HSQC) were as follows: spectral width, 6,000 Hz (^1^H) and 20,742 Hz (^13^C); number of data points, 512 (F1) and 2,048 (F2); number of scans, 256; acquisition time, 0.341 s; and delay time, 2.0 s.

The acquisition parameters of the ^1^H–^13^C constant-time heteronuclear multiple-bond correlation spectroscopy (CT-HMBC)^43^ were as follows: spectral width, 6,000 Hz (^1^H) and 28,902 Hz (^13^C); number of data points, 512 (F1) and 2,048 (F2); number of scans, 256; acquisition time, 0.341 s; and delay time, 2.0 s.

### NMR data processing and signal assignments

All ^1^H NMR spectra were analyzed using FT-NMR data processing software (version 4, Sankyo publishing, Tokyo, Japan) and were manually phased, baseline corrected, and referenced to TSP at 0.00 ppm.

Signal assignments were made according to a previously described method using 2D experiments, a database, and/or spiking experiments^11,12^. The spin systems were confirmed by the ^1^H-^1^H DQF-COSY and ^1^H-^1^H TOCSY NMR spectra. The ^1^H-^13^C HSQC NMR spectrum was used to find correlations between protons and their neighboring carbons, and the ^1^H-^13^C CT-HMBC NMR spectrum was used to confirm the connections of quaternary carbons to protons through two- or three-bond couplings. The signals observed in 1D and 2D NMR spectra were compared with the data provided in several online databases for metabolomics, such as HMDB^44^, the Biological Magnetic Resonance Bank (BMRB)^45^, and the Madison Metabolomics Consortium Database (MMCD)^46^, from which several candidate components were chosen. Finally, signal assignment was accomplished by spiking authentic standard compounds (acetaldehyde, adenosine, ethanol, fumaric acid, glutamic acid, glutamine, phenylalanine, trigonelline, tryptophan, and uridine; special grade; purchased from Wako Pure Chemical Industries, Ltd., Osaka, Japan, or Nacalai Tesque Inc., Tokyo, Japan) into the juice and comparing the spectra of the resulting mixtures with those of unspiked juice.

### STOCSY analysis

The ^1^H NMR spectral data were reduced into 0.04 ppm spectral buckets, and intensity data were normalized for each spectrum and standardized for each bucket. Then, the correlation matrix was calculated according to Cloarec *et al*.^34^. Microsoft Excel and Python with numpy and matplotlib were used for the calculation.

### Multivariate data analysis

The resulting data sets were then imported into SIMCA-P version 13.0 (Umetrics, Umeå, Sweden) for further multivariate data analyses.

Prior to PCA, the bucket data were mean-centered and then scaled using Pareto scaling. Hotelling’s *T*^2^ region, shown as an ellipse in the score plots, defined the 99% confidence interval of the modeled variation. The quality of the model was described by *Rx*^2^ and *Q*^2^ values. *Rx*^2^ was defined as the proportion of variance in the data explained by the model, indicating goodness of fit. *Q*^2^ was defined as the proportion of variance in the data that was predictable by the model, indicating predictability.

OPLS-DA provided the maximum covariance between the measured data (*X*) and the response variable (*Y*). The response variables represent the intensities of the corresponding buckets assigned to separate components. The overall predictive ability of the model was assessed by cumulative *Q*^2^, which represented the fraction of the variation in *Y* that can be predicted by the model^47^.

## Supplementary information

Supplementary Information
