# Supplementary material for: NMR-based metabolic profiling and comparison of Japanese persimmon cultivars
Source: Sci Rep. 2019 Oct 18;9:15011. doi: 10.1038/s41598-019-51489-y (PMC6802078; doi:10.1038/s41598-019-51489-y)
Supplement: Supplementary file 1 — Supplementary Information [file 41598_2019_51489_MOESM1_ESM.pdf]

Supplementary Information for:

NMR-Based Metabolic Profiling and Comparison of Japanese Persimmon Cultivars

Shoraku Ryu,[#] Tomonari Muramatsu,[#] Kazuo Furihata, Feifei Wei, Masanori Koda, Takuya Miyakawa, and Masaru Tanokura*

[†]Department of Applied Biological Chemistry, Graduate School of Agricultural and Life Sciences, The University of Tokyo, 1-1-1 Yayoi, Bunkyo-ku, Tokyo 113-8657, Japan

#Both authors contributed equally to this work

Corresponding Author

*(M.T.) Phone: +81-3-5841-5165. E-mail: amtanok@mail.ecc.u-tokyo.ac.jp

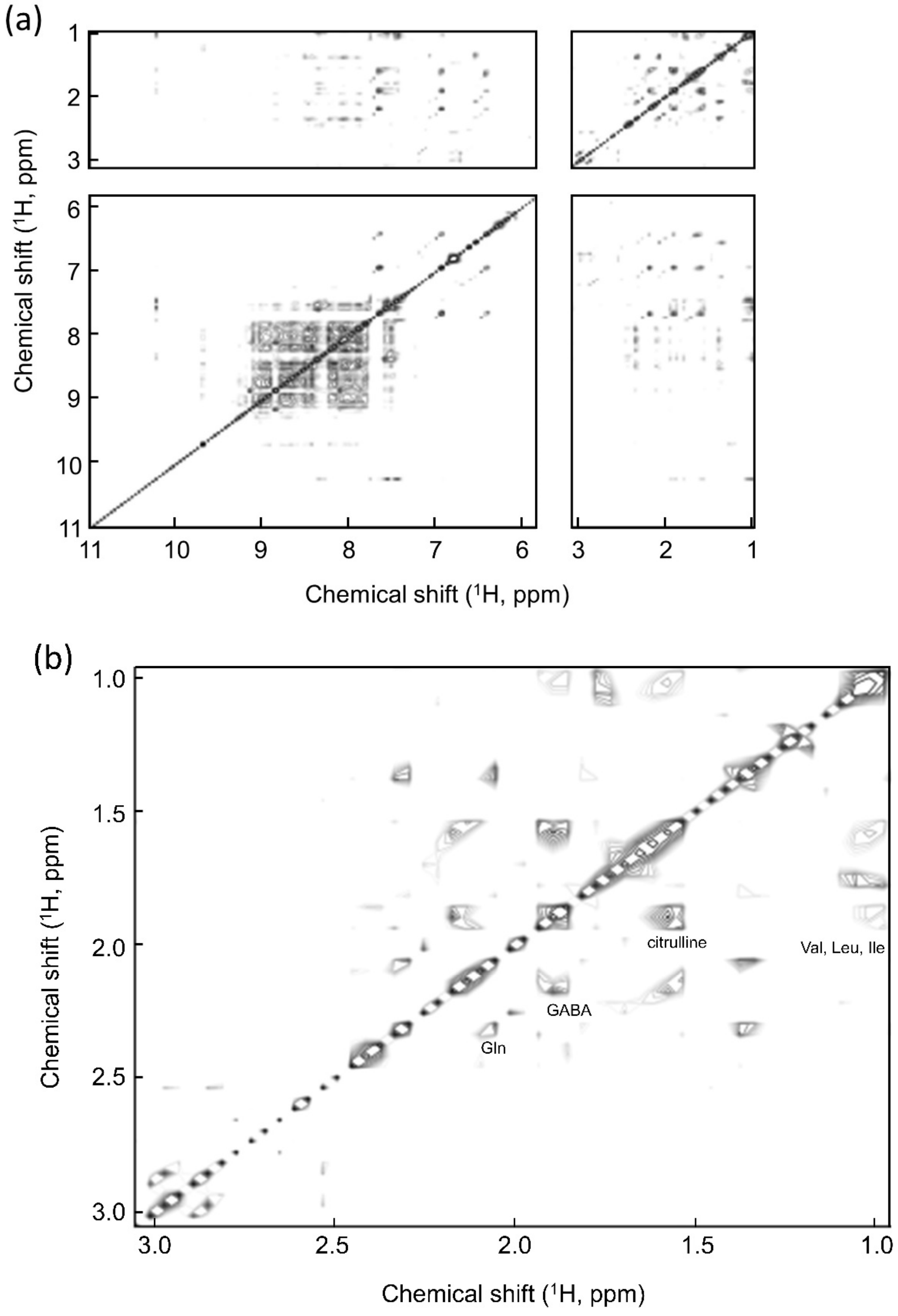

Figure S1. STOCSY analysis of 70 broadband WET NMR spectra using all of the 5 different cultivars (TS, MF, JM, HN, YM) (**(a)** 1-11 ppm, **(b)** 1-3 ppm).

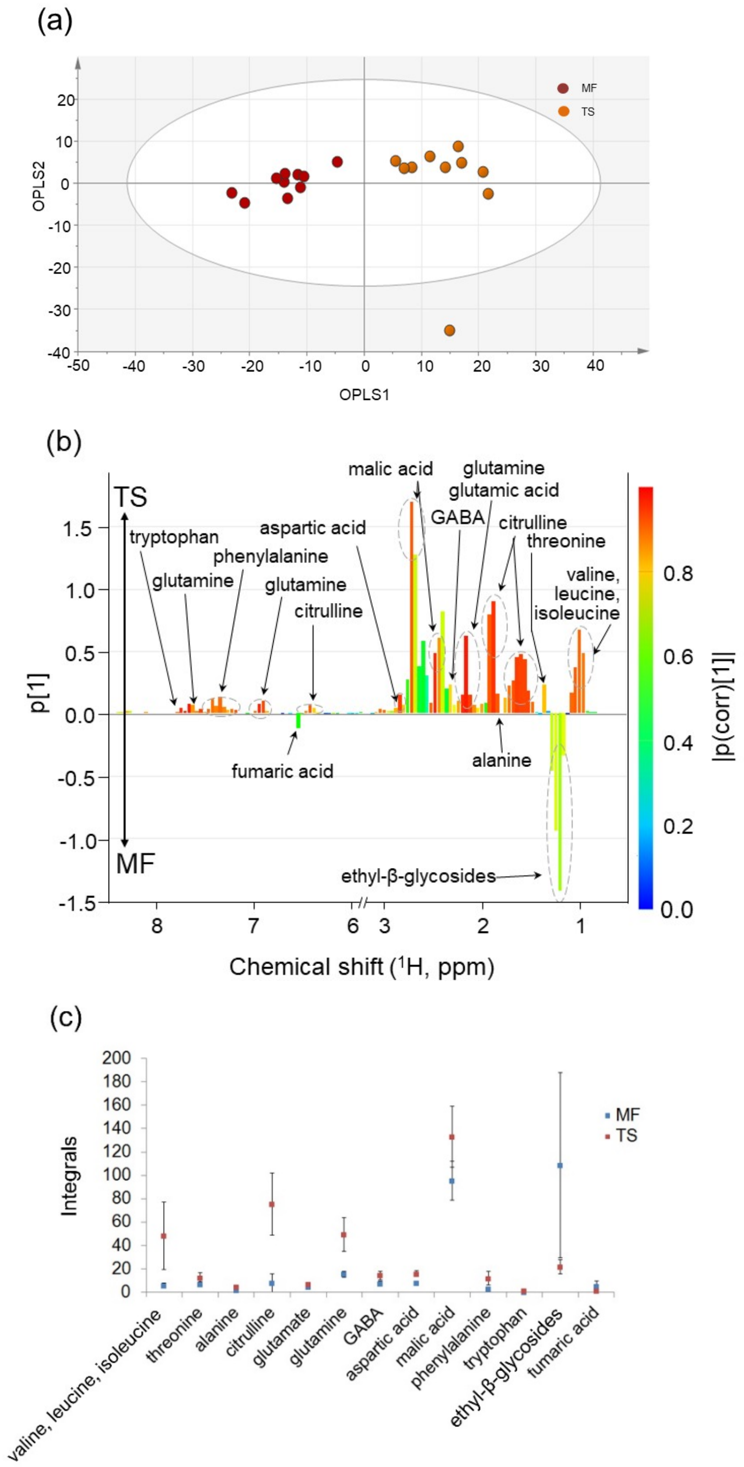

Figure S2. (a) OPLS-DA score plot and **(b)** OPLS-DA loading plot of two nonastringent cultivars (MF and TS). The variances were 56.4% and 19.9% for OPLS1 and OPLS2, respectively. The goodness-of-fit parameters R^2x and R^2y were 0.762 and 0.879, respectively, and the Q^2 value was 0.746. **(c)** The intensities of biomarkers in the MF and TS cultivars. TS and MF represent Taishu and Matsumotowase-Fuyu, respectively. The NMR spectrum of ten persimmon fruits for each of the cultivars was measured individually (n = 10, Error bar: ± standard deviation).

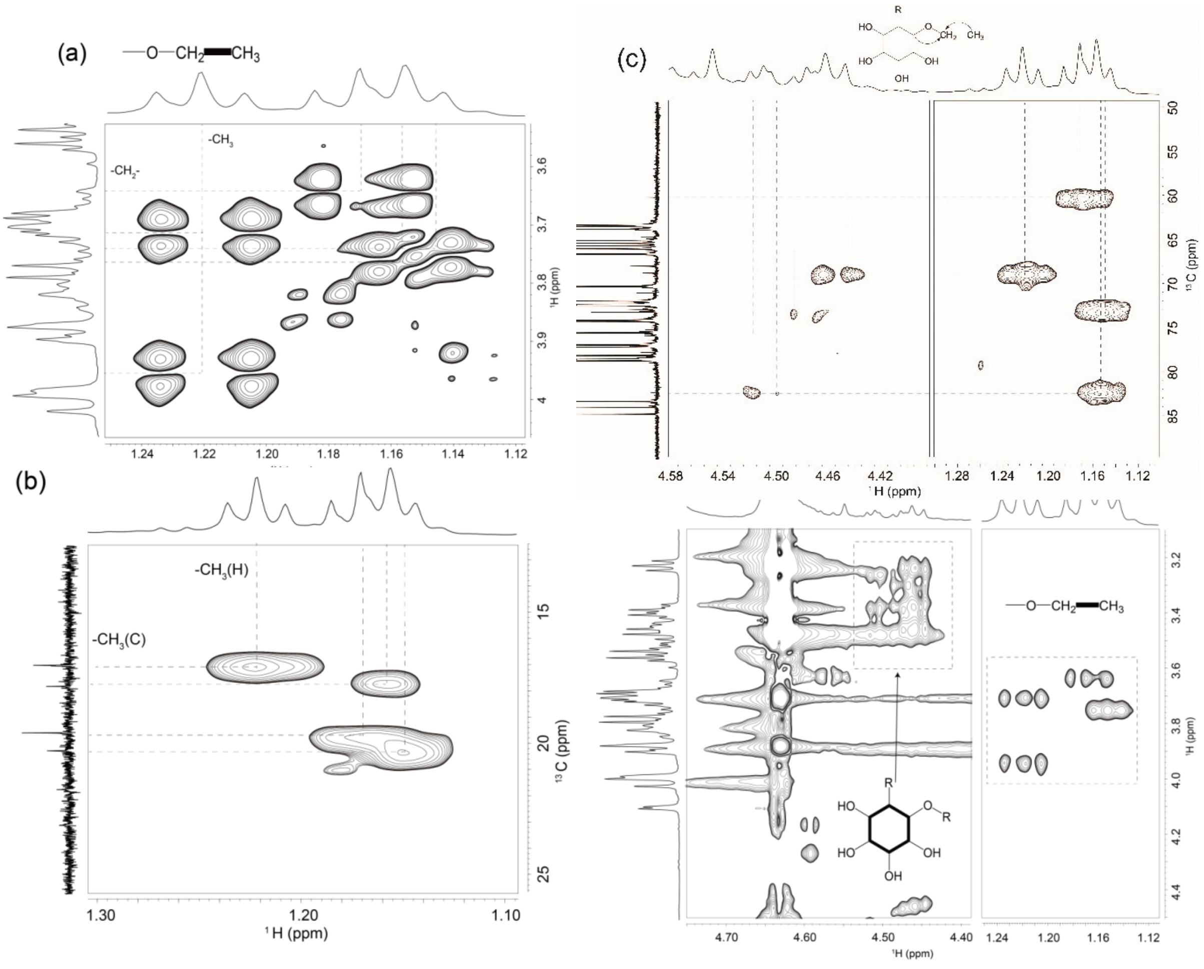

Figure S3. Expansion of the 2D NMR spectra of the MF cultivar with the assignment of ethyl glycosides. **(a)** ^{1}H-^{1}H DQF-COSY spectrum. The bolded bonds in the chemical structure indicate possible correlations in the ^{1}H-^{1}H DQF-COSY spectrum. **(b)** ^{1}H-^{13}C HSQC spectrum. **(c)** ^{1}H-^{13}C CT-HMBC spectrum. The arrows in the chemical structure indicate possible correlations in the ^{1}H-^{13}C CT-HMBC spectrum. **(d)** ^{1}H-^{1}H TOCSY spectrum. The bolded bonds in the chemical structure indicate possible correlations in the ^{1}H-^{1}H TOCSY spectrum.

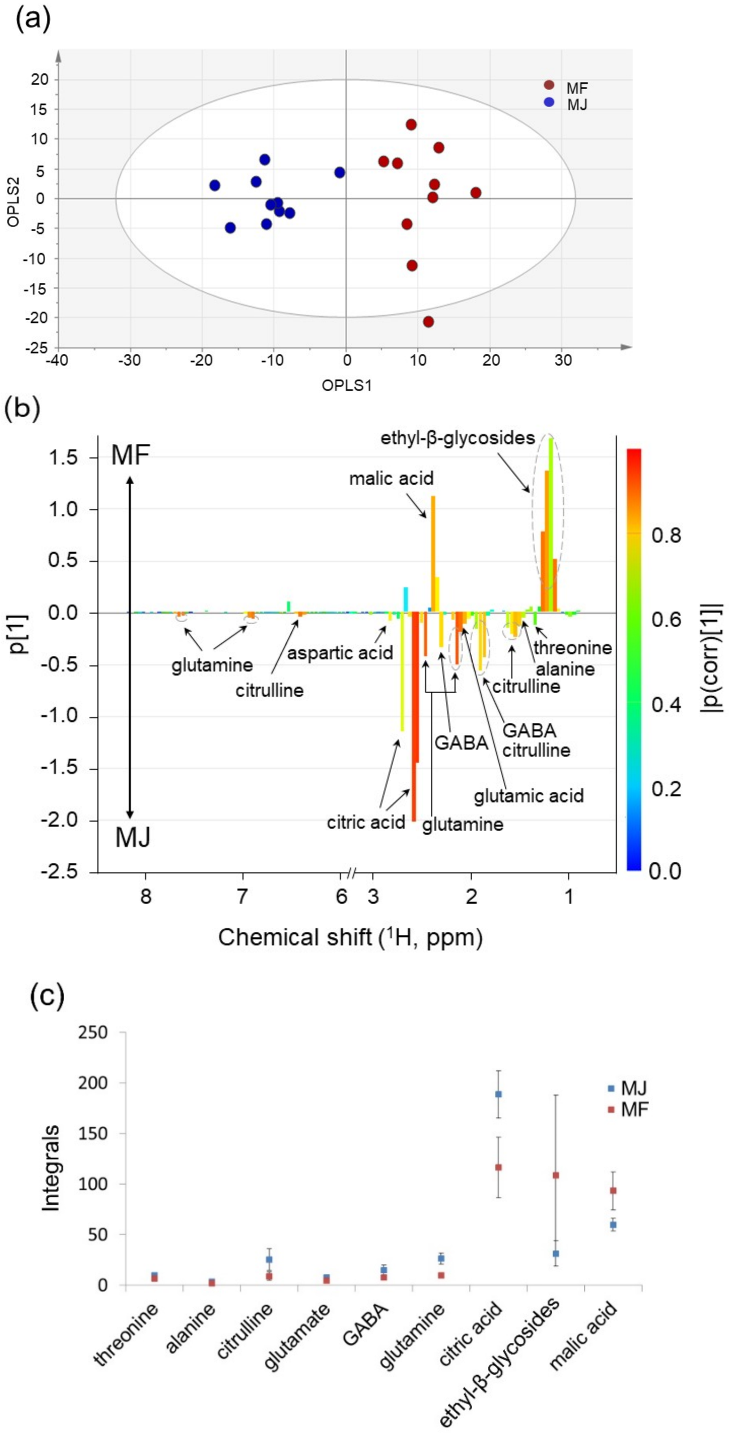

Figure S4. (a) OPLS-DA score plot and **(b)** OPLS-DA loading plot of two nonastringent cultivars (MF and MJ). The variances were 49.5% and 19.3% for OPLS1 and OPLS2, respectively. The goodness-of-fit parameters R^2x and R^2y were 0.688 and 0.878, respectively, and the Q^2 value was 0.730. **(c)** The integral intensities of biomarkers in the MF and MJ cultivars. MF and MJ represent Matsumotowase-Fuyu and Maekawa Jiro, respectively. The NMR spectrum of ten persimmon fruits for each of the cultivars was measured individually (n = 10, Error bar: ± standard deviation).

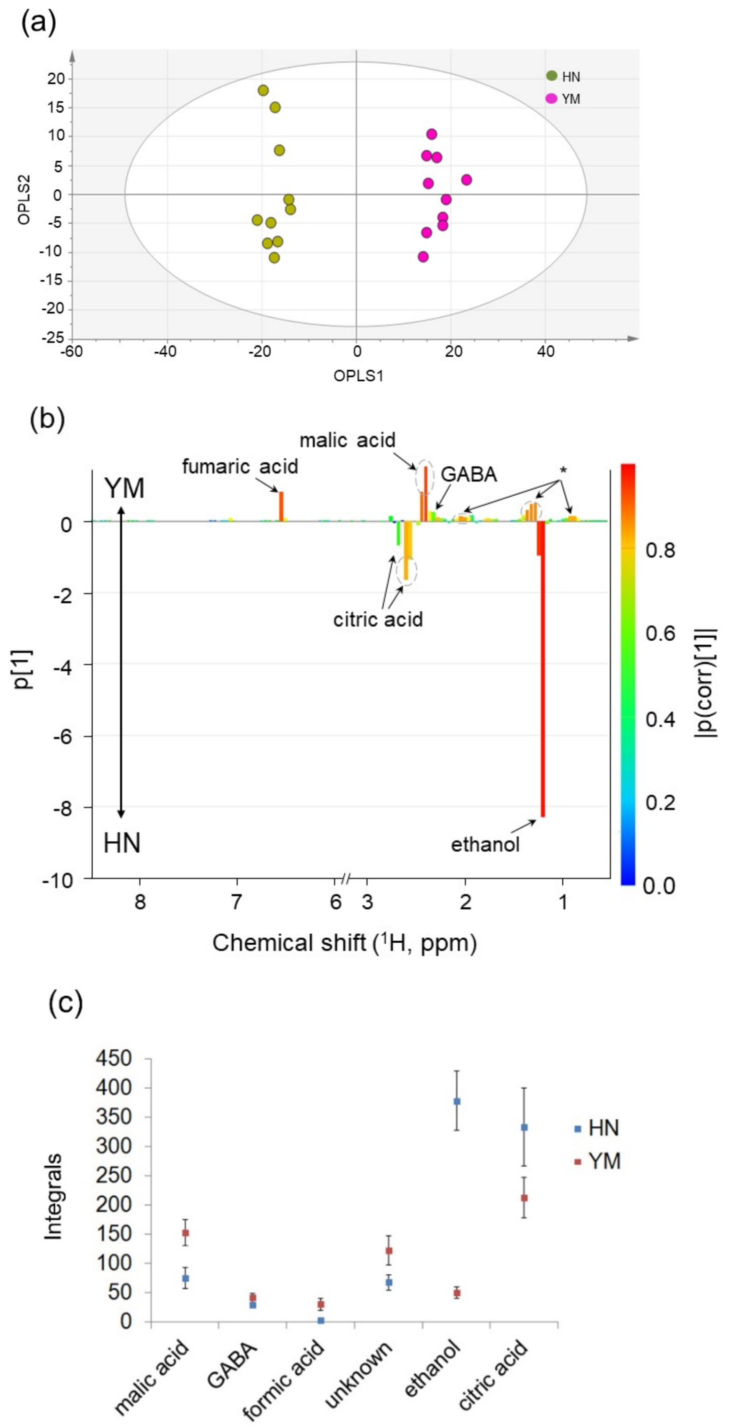

Figure S5. (a) OPLS-DA score plot and **(b)** OPLS-DA loading plot of two astringent cultivars (HN and YM). The variances were 64.9% and 14.3% for OPLS1 and OPLS2, respectively. The goodness-of-fit parameters R^2x and R^2y were 0.791 and 0.981, respectively, and the Q^2 value was 0.974. **(c)** The integral intensities of biomarkers in the HN and YM cultivars. HN and YM represent Hiratanenashi and Yotsumizo, respectively. Asterisks denote unknown components. The NMR spectrum of ten persimmon fruits for each of the cultivars was measured individually ($n = 10$, Error bar: ± standard deviation).
